# Long-term improvements in executive functions after frontal-midline theta neurofeedback in a (sub)clinical group

**DOI:** 10.3389/fnhum.2023.1163380

**Published:** 2023-06-09

**Authors:** Diede Smit, Cecilia Dapor, Janneke Koerts, Oliver M. Tucha, Rene J. Huster, Stefanie Enriquez-Geppert

**Affiliations:** ^1^Department of Clinical and Developmental Neuropsychology, University of Groningen, Groningen, Netherlands; ^2^Research School of Behavioural and Cognitive Neurosciences, University of Groningen, Groningen, Netherlands; ^3^Department of Psychology and Cognitive Science, University of Trento, Trento, Italy; ^4^Department of Psychiatry and Psychotherapy, University Medical Center Rostock, Rostock, Germany; ^5^Department of Psychology, National University of Ireland, Maynooth, Irleand; ^6^Department of Psychology, University of Oslo, Oslo, Norway; ^7^Department of Biomedical Sciences of Cells and Systems, University Medical Center Groningen, Groningen, Netherlands

**Keywords:** neurofeedback, executive (dys)functions, subjective cognitive complaints, frontal-midline theta, working memory updating, set-shifting, conflict monitoring, response inhibition

## Abstract

Impairments in executive functions (EFs) are common across disorders and can greatly affect daily functioning. Frontal-midline (FM) theta neurofeedback (NF) has been shown effective in enhancing EFs in healthy adults, prompting interest in exploring its potential as an alternative treatment for EFs in (sub)clinical samples. This study aims to determine the effects of FM theta NF on EFs in a sample of 58 adults (aged 20–60 years) with pronounced subjective EF complaints in daily life. Using a pre/post/follow-up design with a sham NF group, the present study assessed upregulation of FM theta in an eight-session individualized FM theta NF training and its immediate and long-term transfer effects on objective and subjective measures of EFs. These included behavioral performance on EF tasks assessing working memory updating (N-back task), set-shifting (Switching task), conflict monitoring (Stroop task), and response inhibition (Stop-signal task), as well as FM theta power during these tasks, and subjective EFs in daily life (BRIEF-A). The results indicate that there are only differences in FM theta self-upregulation between the NF group and sham group when non-responders are excluded from the analysis. Regarding behavioral transfer effects, NF-specific improvements are found in working memory updating reaction time (RT) and conflict monitoring RT variability at 6-month follow-up, but not immediately after the NF training. The effects on FM theta power during the EF tasks and subjective changes in EFs in daily life were not specific to the NF training. As a next step, research should identify the best predictors to stratify NF training, as well as explore ways to improve NF responsiveness, for instance by increasing neuroplasticity.

## 1. Introduction

Impairments in executive functions (EFs) can be regarded as a transdiagnostic feature in many psychiatric disorders (Snyder et al., 2015; Abramovitch et al., 2021), and are associated with a range of health problems such as reduced daily functioning, poorer quality of life, and depressive symptoms (Vaughan and Giovanello, 2010; Letkiewicz et al., 2014; Zhang et al., 2021). EFs refer to a set of separate but interrelated higher cognitive (control) processes (Friedman and Miyake, 2017), including working memory updating, set-shifting, conflict monitoring, and response inhibition (Miyake et al., 2000; Enriquez-Geppert et al., 2010). Given the crucial role of EFs in enabling independent, flexible, and goal-oriented behavior in everyday life (Diamond, 2013), and their frequent impairment in various disorders, there is a need for effective treatment approaches to improve them. Neurofeedback (NF) has shown promise in effectively boosting EFs in healthy adults (Viviani and Vallesi, 2021), leading to the question of whether these effects can be replicated in (sub)clinical populations.

Neuroscientific treatment approaches such as NF, transcranial alternating current stimulation (tACS), and transcranial direct current stimulation (tDCS) aim to directly target underlying brain mechanisms of cognition or clinical symptoms. NF is particularly promising as it is an active self-neuromodulation approach that includes learning mechanisms (Enriquez-Geppert et al., 2017; Sitaram et al., 2017) and neuroplastic effects (Ros et al., 2014), and thus potentially leads to more sustainable long-term effects (e.g., Van Doren et al., 2019). NF is a non-invasive technique that employs a brain-computer-interface to record brain activity, analyze it, and feeds selected brain features back to the participant in real-time (Marzbani et al., 2016). This real-time feedback serves as a guiding mechanism for the participant to modulate and regulate those brain features in the desired direction with the end goal of influencing cognition or clinical symptoms (e.g., Enriquez-Geppert et al., 2013a).

A systematic review by Viviani and Vallesi (2021) demonstrated that NF studies applying a frontal midline (FM) theta protocol were most successful in targeting EFs. Theta oscillations (4–8 Hz) recorded at the FM region are considered crucial for EFs. During events require the engagement of EFs, theta oscillations are increased with a main generator in midcingulate cortex (MCC; Cavanagh and Frank, 2014; Eisma et al., 2021). The MCC is an important hub within the superordinate fronto-cingulo-parietal network (Niendam et al., 2012). Increased theta oscillatory power has been found to be linked to stronger neuronal spike-field coupling in the theta band (Helfrich and Knight, 2016). Furthermore, this increase is associated with better performance on tasks requiring EFs (e.g., Nigbur et al., 2011; Itthipuripat et al., 2013; Cooper et al., 2017; Eschmann et al., 2018). Based on these findings, four studies have assessed a NF protocol specifically targeting FM theta oscillatory power to enhance EFs in healthy young and older adults (Wang and Hsieh, 2013; Enriquez-Geppert et al., 2014a; Brandmeyer and Delorme, 2020; Eschmann and Mecklinger, 2022). These studies showed a significantly larger increase in FM theta power for the NF group as compared to an active control group after the NF training, and most importantly behavioral transfer effects on proactive processes of EFs (for the distinction between proactive and reactive processes the article by Braver, 2012).

In the search for an effective treatment approach for executive dysfunctions, the current study investigates the effects of a FM theta NF training in individuals with pronounced self-reported EF complaints in daily life, independent of whether or not they have a psychiatric diagnosis. The focus on this (sub)clinical group is considered a next step in evaluating the efficacy of FM theta NF as a treatment option for individuals with subjectively experienced impairments of EFs beyond its known effects in healthy participants. This study will assess the self-regulatory ability of FM theta through NF, as well as its immediate and long-term effects on objective measures of EFs and self-reported EFs. These results will contribute to the ultimate goal of developing a transdiagnostic NF training that can be used as a standalone treatment in a clinical context, but also in combination with other therapies.

## 2. Materials and methods

### 2.1. Recruitment and inclusion criteria

Participants were recruited via advertisements on social media and completed the Behavior Rating Inventory of Executive Function–Adult Version (BRIEF-A; Roth et al., 2005) to assess their eligibility. EF complaints were operationalized as a score in 90th percentile or higher (i.e., very high/impaired range) on the BRIEF-A total score (≥128) or on at least one of the subscales: Working memory (≥15), Shift (≥12), Task monitor (≥12), or Inhibit (≥15). These subscales are thought to represent the four EFs: working memory updating, set-shifting, conflict monitoring, and response inhibition, respectively (Roth et al., 2005). For this study, individuals with a severe neurological disorder (such as a brain tumor) or psychiatric disorder (such as schizophrenia) significantly affecting daily functioning were excluded. The study allowed the use of medication to not withhold medication from participants for an extended period of time and to be able to generalize results.

### 2.2. Participants

**FIGURE 1:**
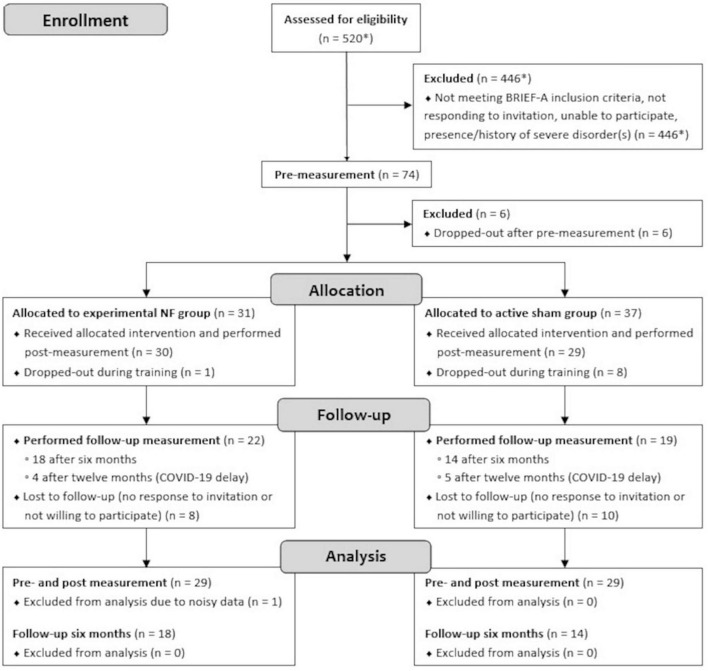
CONSORT flow diagram of measurements performed in the lab. *Estimation.

A convenience sample of 58 Dutch speaking adults with pronounced self-reported EF complaints in daily life participated in this study. Participants were pseudo-randomly assigned to either the NF group (*n* = 29) or the sham group (*n* = 29) to dissociate NF-specific effects from other non-specific effects. The groups were matched as closely as possible in terms of age, gender, education level, and psychiatric disorders. Education level was rated on an eight-level scale and classified into low (i.e., primary education [1] or preparatory secondary vocational education [2]), intermediate (i.e., secondary vocational education [3], senior general secondary education [4], or pre university education [5]), or high (i.e., higher vocational education [6], university bachelor [7], or university master [8]). The CONSORT flow diagram of the study is presented in Figure 1. Prior to the start, information about voluntary participation in the study was provided and all participants gave written consent. The study was single-blinded; participants were only informed of assignment to one of two different NF training protocols. The majority of research assistants performing the NF sessions were aware of the group assignments due to its visibility in the used NF software. Instructions and interactions with participants were kept as similar as possible between both groups. The study protocol was approved by the Ethical Committee of the Behavioral and Social Science Faculty of the University of Groningen, Netherlands, and conducted in accordance with the Declaration of Helsinki.

### 2.3. Procedure and materials

Data collection took place in a sound-attenuated EEG lab at the Heymans institute at the University of Groningen, Netherlands. Participants in both the NF and sham group followed the same training schedule, which consisted of a pre-measurement, eight NF training sessions, and a post-measurement, all completed within approximately three consecutive weeks (*M* = 19 days, *SD* = 5.0). Six months after the NF training, participants were invited for a follow-up measurement. Due to COVID-19 restrictions, 30% of the sample did not complete this 6-month follow-up measurement. Participants who were unable to come to the lab for the follow-up measurement were asked to complete the BRIEF-A questionnaire online from home. Nine participants had their follow-up measurement 12 months post-training. After completing the follow-up measurement, participants were asked to guess which group they belonged to and were then debriefed about their group assignment.

#### 2.3.1. Pre-, post-, and follow-up measurements

The measurements (pre-, post-, and follow-up) had a consistent structure and included questionnaire(s), a resting state EEG recording, and four computerized EF tasks. Each measurement session took approximately 120 to 150 min to complete. On average, the 6-month follow-up measurement was performed 203 days (*SD* = 30.6, *n* = 32) after the post-measurement, and the 12-month follow-up measurement 342 days (*SD* = 20.1, *n* = 9) after the post-measurement.

##### 2.3.1.1. Questionnaires

In the pre-, post-, and follow-up measurements, participants completed the BRIEF-A questionnaire while the EEG cap was set. The BRIEF-A assesses the frequency of certain EF problems in daily life on a three-point scale over the past month (Roth et al., 2005). In this study, the total score (i.e., combination of nine subscales) and the subscales Working Memory, Shift, Task Monitor, and Inhibit were used.

At post-measurement, two additional questionnaires were administered to assess the presence of depressive symptoms and ADHD symptoms during the NF training. The Beck Depression Inventory II (BDI-II) was used to assess depressive symptoms over the past 2 weeks (Beck et al., 1996). For 21 items, referring to specific symptoms, participants had to choose one of four statements that best applied to them. A score of <13 is considered minimal, 14–19 mild, 20–28 moderate, and >29 severe. The Self-report Questionnaire on Attention problems and Hyperactivity for adulthood and childhood (Dutch: Zelf-rapportage Vragenlijst over Aandachtsproblemen en Hyperactiviteit voor volwassenheid en kindertijd [ZVAH]) was used to assess ADHD symptoms (Kooij et al., 2005). Participants were asked to rate on a four-point scale how often they displayed certain behaviors in the past 6 months and during childhood. The adulthood version was used, which assesses nine criteria for attentional symptoms and nine for hyperactivity. A score of four or more out of nine criteria was used as a cut-off (Kooij et al., 2005).

##### 2.3.1.2. EF tasks

**FIGURE 2:**
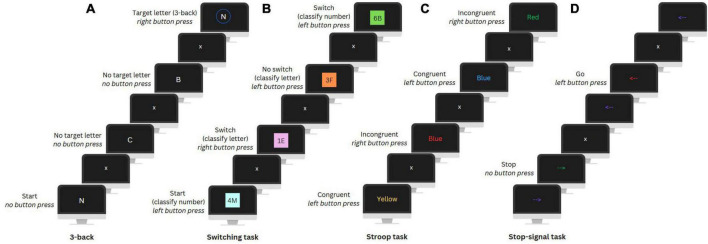
Visual illustration of the 3-back (update) condition of the N-back task **(A)**, Switching task **(B)**, Stroop task **(C)**, and Stop-signal task **(D)**.

After completing the questionnaires, participants completed an 8-min EEG resting state measurement (eyes open and closed, not used in this study), followed by four computerized EFs tasks while their EEG was being measured: the N-back, Switching, Stroop, and Stop-signal task (Figure 2).

The N-back task was used to assess working memory updating and includes a No update (0-back) and an Update condition (3-back). In the No update condition, participants had to press a button when a letter matched a target letter presented at the beginning of a letter sequence. In the Update condition, participants were instructed to press a button when a letter matched the letter presented three positions earlier in the sequence. There were ten Update sequences and nine No update sequences with 24 trials (i.e., letters) per sequence and eight target letters. Each trial lasted 2000 ms and included a fixation cross and the letter presentation.

A Switching task was utilized to assess set-shifting. In this task, participants were presented with number-letter pairs on a colored background and had to classify either the number or letter. In the first two unmix blocks, participants had to categorize only the number (even or odd) or letter (vowel or consonant) to get familiar with the task. In the third mixed block, classification of either the number or letter was based on the background color. A Switch condition required a switch between number and letter classification and in a No switch condition the classification category remained the same as in the previous trial. The mixed block included a total of 234 trials of which 70 were switch trials. Each trial lasted 3000 ms and included a fixation cross and the presentation of the letter-number pair, followed by a filler period.

The Stroop task was used to assess conflict monitoring. Participants were presented with color words in either the same or different color as the word (i.e., the Congruent and Incongruent condition, respectively) and had to indicate the color. For both conditions there were 72 trials, each lasting an average of 2700 ms. Trials included a fixation cross, color word presentation, and an inter-trial interval. Feedback on performance was automatically given after every sixteen trials to encourage fast and accurate responses.

The Stop-signal task was used to assess response inhibition. In this task, participants were presented with arrows pointing left or right that changed color during their presentation and had to press the corresponding button (i.e., Go condition). A change to a specific color indicated to inhibit their motor response (i.e., Stop condition). For this, the timing of the color change was adjusted dynamically, adding 50 ms after every second correct or subtracting 50 ms after an incorrect stop trial, to ensure that participants would stop their response in 50% of the trials. There were 300 trials, including 100 Stop condition trials. Each trial lasted 2000 ms and included a fixation cross, an arrow presentation (adjusted by a stop signal delay), and again a fixation cross. For more details on the four tasks see Smit et al. (2023).

There were two lists, with a different order of the tasks and stimulus-response assignment. Prior to each task, written instructions and a short practice were provided to familiarize participants with the task. Participants were required to respond to stimuli using a button box with two answering options. During the completion of the tasks, participants were instructed to maintain still and reduce blinking to a minimum. Breaks were given between tasks upon request. Each task included a condition that required EFs (i.e., Update, Switch, Incongruent, Stop) and a control condition (i.e., No update, No switch, Congruent, Go), and lasted between 8 and 9 min. The EF tasks were administered in a sound-attenuated room using Presentation software (Neurobehavioral Systems version 14.8).

##### 2.3.1.3. EEG recordings and pre-processing

All EEG measurements were carried out by trained researchers and assistants with a background in (neuro)psychology. EEG was recorded using a 64 Ag/AgCl electrodes Waveguard connect cap, an average reference Twente Medical Systems International BV (TMSi) REFA amplifier, and Openvibe recording software (Renard et al., 2010). Electrodes were placed according to the extended version of the international 10–20 system, with additional vertical and horizontal electrodes on the dominant eye for recording the electro-oculogram (EOG). Electrode impedances were regularly checked to ensure they were below 10 kΩ. The amplifier provided 24-bit resolution EEG data at a sampling rate of 256 Hz.

All offline preprocessing was performed in MATLAB version R2019B using the EEGLAB toolbox (Brunner et al., 2013). First, the data was filtered using a low-pass filter (40 Hz) and a high-pass filter (0.1 Hz), down-sampled to 250 Hz, and re-referenced to two mastoid electrodes. Next, independent component analysis using the *Runica* algorithm was applied for removal of eye blinks and horizontal eye movements. The continuous EEG data was then epoched from −1250 to 1250 ms relative to stimulus onset (e.g., presentation of color word in the Stroop task or target letter in the N-back task). Rest-artifact correction was performed in a semi-automated procedure in which trials exceeding a threshold of 60 μV were flagged and visually inspected. Electrodes with excessive noise-related fluctuations (e.g., due to impedance increase) were interpolated. For each task, a maximum difference of ten epochs between the two conditions was achieved by randomly removing correct trials for each participant. The minimum number of epochs for a condition was 27. Table 1 shows the final sample sizes included in the EEG analyses.

**TABLE 1:** Sample sizes per measurement, group, and task included in the EEG analyses.

	Pre-measurement		Post-measurement		6-month follow-up*		12-month follow-up*	
**Task**	**NF (*n* = 29)**	**Sham (*n* = 29)**	**NF (*n* = 29)**	**Sham (*n* = 29)**	**NF (*n* = 18)**	**Sham (*n* = 14)**	**NF (*n* = 4)**	**Sham (*n* = 5)**
N-back	27	27	27	28	17	14	4	5
Switching	24	23	28	26	18	13	4	5
Stroop	28	28	29	29	18	14	4	5
Stop-signal	25	23	26	26	13	13	3	3

*Due to COVID-19 regulations, not all participants could perform the 6-month follow-up measurement. Therefore, nine participants did a 12-month follow-up measurement instead.

Next, event-related spectral perturbations (ERSPs) were calculated using the *newtimef* function, which transforms the data to represent log-transformed changes in power in dB relative to the baseline (Delorme and Makeig, 2004). For the time-frequency decomposition a Morlet wavelet transform was used with an increasing number of cycles with increasing frequencies (range: 2–30 Hz, starting with one cycle at two Hz and increasing by 0.5 cycles per one Hz increment, ending with fifteen cycles at 30 Hz). To visualize changes in power relative to activity before stimulus onset, the mean power across trials was divided by frequency-specific baseline values for each frequency. The mean ERSP values were calculated for the FM region using electrodes Fz, FC1, FCz, FC2, and Cz.

#### 2.3.2. Individualized eight session NF training

The FM theta NF training was personalized for each participant based on the individual theta peak (ITP), which can vary significantly between individuals but has high intra-individual stability (Näpflin et al., 2008). The ITP was based on the EEG data collected during the four EF tasks in the pre-measurement. For each task condition requiring EFs, the ITP was identified in the ERSPs for the FM region, and the mean peak across the four tasks was calculated. The mean peak ± 1 Hz was used for the NF training. The feedback signal was based on the EEG data recorded at five electrodes at the FM: Fz, FC1, FCz, FC2 and Cz. Recordings were online referenced to the nose, and Fp1 and Fp2 were used for the EOG. EEG signals were read out in real-time and processed by the Matlab-based software NeuroSuite 2.0. The sampling rate was 500 Hz.

**FIGURE 3:**
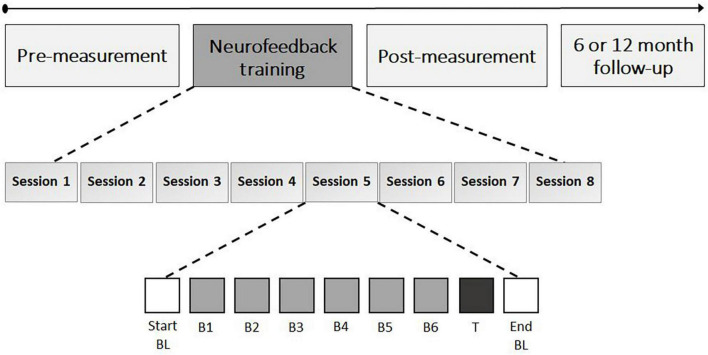
Neurofeedback training schedule. The neurofeedback training consisted of eight sessions with each 35 min of FM theta upregulation time; 5 min per block (i.e., neurofeedback B1 to B6 and transfer block [T]). The start and end baseline (BL) also took 5 min each and assessed resting state EEG.

The eight NF sessions consisted of an EOG calibration, start baseline, six NF blocks, transfer block, and end baseline, with self-paced breaks in between (see Figure 3). EOG calibration (3 min) was used to identify artifacts (e.g., eye blinks). A manual threshold was first set, and two-second epochs centered around the artifact peaks were extracted. The mean and SD of all epoch values exceeding 0.75 times the threshold were then calculated, and the final threshold was determined as the mean minus the SD. During online processing in subsequent blocks, incoming data underwent detrending and rectification, and an epoch was flagged as an artifact if it exceeded the final threshold. During the start baseline (5 min), resting state activity was measured and participants were instructed to rest without engaging in any cognitive process or forcing a state of relaxation. During the NF blocks (5 min each), participants were instructed to actively increase FM theta power relative to the start baseline by using mental strategies. All participants in both groups were presented with a list of mental strategies, including mental acts (e.g., mental arithmetic, mental rotations of objects), relaxation (e.g., focus on breathing), imagining emotions (positive or negative), retrieving memories (e.g., about family, holidays), auditory strategies (e.g., imagining music), cheering for a red square, imagining movement or activities (e.g., foot movement or practicing sport), and remembering or imagining nature (e.g., rain, sunset), or daily activities (e.g., cooking, shopping). Additionally, they were encouraged to test their own strategies and use the most effective ones. A colored square on the computer screen provided real-time feedback on the effectiveness of the strategy, with the color ranging from highly saturated blue (i.e., below 2.5% of the amplitude range) to gray in the middle as an anchor to highly saturated red (i.e., above 97.5% of the amplitude range) in 21 color steps. The feedback signal was updated every 250 ms based on a two second sliding window that captured the incoming data. Fast-Fourier Transform was used to calculate the amplitude of the individually determined theta-band. The participants’ goal was to color the square as red as possible, indicating an increase in FM theta power relative to the baseline, while blue represented a decrease. A gray square indicated no difference in FM theta amplitude or the detection of an artifact. The color of the feedback signal was scaled such that a maximal saturation corresponded to theta amplitudes ± 2 SD from the mean of the baseline.

Participants in the sham group received a replay of feedback from a matched participant in the NF group for the same session and block, in order to provide both groups with similar visual feedback. To enhance the credibility of the feedback in the sham group, participants received real feedback on their own (eye) artifacts (i.e., gray square). After the six NF blocks, a transfer block (5 min) was conducted in which participants were again asked to apply mental strategies to increase FM theta power, but without visual feedback (i.e., the square remained gray). After each NF block and transfer block participants were asked to write down the mental strategies they used and to evaluate their effectiveness on a seven-point scale. The sessions concluded with a resting-state end baseline measurement (5 min) in which no strategies were required and the instructions were the same as for the start baseline. During both the start- and end baseline measurements, the square changed colors with a random gradient to provide visual stimulation similar to the NF blocks. Finally, in each session, participants were asked to self-evaluate their motivation for participating in the study, their level of commitment to the study, and their perception of difficulty, using a seven-point Likert scale. Each NF session took approximately 75 min to complete.

### 2.4. Statistical analysis

#### 2.4.1. NF training effects

As a first step, the amplitudes in all blocks were normalized to the overall power (1–30 Hz) in four individualized frequency bands: theta (ITP ± 1), delta (ITP – 3.5–1.5 Hz), alpha (ITP + 3–5 Hz), and beta (ITP + 7–24 Hz). Subsequently, two learning indexes were used to evaluate the effects of the individualized FM theta NF training on the upregulation of theta. Next, a within-session baseline correction was applied, in which the increase in FM theta was calculated as the difference in mean amplitude between a specific block and the baseline of that respective session (e.g., mean amplitude NF block 1 in session 1—mean amplitude start baseline in session 1). This approach minimizes the effects of inter-individual differences in FM theta amplitude and measurement variability across sessions.

For the first learning index (Learning Index 1), the changes in FM theta amplitude from session to session were assessed. For each session, the mean relative theta amplitude across the six NF blocks was calculated. Training effects were analyzed using repeated measures (RM) ANOVA with SESSION (1–8) as the within-subject factor and GROUP (NF vs. sham) as the between-subjects factor. With the second learning index (Learning Index 2), the dynamical changes within sessions were assessed (Enriquez-Geppert et al., 2014b). For each block, the mean relative theta amplitude across all sessions was calculated. Effects were analyzed using RM ANOVA with within-subject factor BLOCK (start baseline, NF blocks 1–6, transfer block, and end baseline) and between-subjects factor GROUP (NF vs. sham). To determine the specificity of the FM theta NF training, the same analyses were performed for delta, alpha, and beta.

#### 2.4.2. Classification of responders and non-responders

Previous research on NF has demonstrated that a portion of participants seems unable to regulate their own brain activity (Alkoby et al., 2018; Haugg et al., 2021). Therefore, we conducted an additional analysis to assess FM theta NF learning in the responders. This distinction is crucial in the context of clinical applications, as it has the potential to inform about a personalized treatment approach where only individuals who demonstrate a positive response to NF would receive it. Such a stratification could significantly enhance the overall effectiveness of the treatment, as well as improve patient outcomes. Participants were classified as responders or non-responders to NF based on the regression slope [i.e., negative slope (≤0) = non-responders and positive slope (>0) = responders] across seven values: the mean relative amplitude for the start baseline (i.e., zero) and the six separate NF blocks averaged across all sessions (i.e., Learning Index 2). This approach takes into account potential changes in theta in the start baseline over the sessions. For theta, RM ANOVAs were conducted for the two learning indices, using GROUP as the between-subjects factor (NF responders vs. sham). In addition, descriptive statistics were compiled for both responders and non-responders to gain insight into the reasons for any differences in theta upregulation.

#### 2.4.3. Testing the credibility of the sham group

To assess the credibility of the sham NF and ensure that the participants were unaware of their group assignment, a chi-square test of independence was conducted. Additionally, RM ANOVA was performed on the dependent variables motivation, commitment, and perceived difficulty with SESSION (1–8) as the within-subject factor and GROUP (NF vs. sham) as the between-subjects factor. Missing data (i.e., eight items were not filled in) was imputed using the mean of the session before and after for the same participant. Finally, descriptive statistics were compiled to qualitatively determine if there were differences between participants who completed the follow-up measurement and those who dropped out after the post-measurement.

#### 2.4.4. Behavioral transfer effects

To evaluate the transfer effects of the NF training on behavioral EF performance, the mean accuracy (AC), reaction time (RT), and RT variability (RTV) were calculated for the correct trials of the EF tasks at the pre-, post-, and follow-up measurement. For the Stop-signal task, the stop-signal reaction time (SSRT) was estimated (Logan and Cowan, 1984). Analyses were only performed on conditions requiring EFs (i.e., Update, Switch, Incongruent, and Stop) to test our hypotheses and reduce the number of statistical tests. To assess the immediate transfer effect of the NF training, RM ANOVA was performed for AC, RT, and RTV with TIME (pre vs. post) as the within-subject factor and GROUP (NF vs. sham) as the between-subjects factor. To assess the long-term effects after 6 months, RM ANOVAs were repeated with TIME (pre vs. follow-up) as the within-subject factor and GROUP (NF vs. sham) as the between-subjects factor. Descriptive data was provided for participants who completed the 12-month follow-up (*n* = 9).

#### 2.4.5. Correlations between self-regulation of FM theta and behavioral changes in EFs

To explore the association between upregulation success in the NF training and change in behavior (i.e., AC, RT, and RTV) immediately after the NF training and in the long-term, Pearson correlation coefficients were calculated. Upregulation success was quantified as the average of all NF blocks across the eight sessions relative to their baseline and changes in behavior by the differences between the scores at the pre-measurement and the post- or 6-month follow-up measurement.

#### 2.4.6. Transfer effects to FM theta during EF tasks

To evaluate the transfer effects of the NF training on FM theta power during the four EF tasks, mean ERSP values were calculated for the theta frequency range (4–8 Hz) from 100 to 500 ms after stimulus onset in electrodes Fz, FC1, FCz, FC2, and Cz. This time range was chosen because of the known engagement of EFs recruitment during this period. Individual time and frequency picking was conducted within the specified range. Subsequently, we computed the average FM theta power by considering a time interval of ± 50 ms and a frequency range of ± 1 Hz around the identified peak. The data was averaged for each participant, task, and condition. To examine the immediate transfer effect of the NF training on FM theta power, a RM ANOVA was conducted for each task condition requiring EFs (i.e., Update, Switch, Incongruent, and Stop) with TIME (pre vs. post) as the within-subject factor and GROUP (NF vs. sham) as the between-subjects factor. To assess the long-term effect after 6 months, RM ANOVAs were conducted again for the four task conditions with TIME (pre vs. follow-up) as the within-subject factor and GROUP (NF vs. sham) as the between-subjects factor. Descriptive data is provided for participants who completed the 12-month follow-up (*n* = 9).

#### 2.4.7. Transfer effects to EFs in daily life

The effects of NF training on subjective EFs in daily life were evaluated using RM ANOVA. The outcome variables were the BRIEF-A total score and the subscales Working memory, Shift, Task Monitor, and Inhibit. To examine the immediate transfer effect, the within-subject factor was TIME (pre vs. post) and the between-subjects factor was GROUP (NF vs. sham). To assess the long-term effect after 6 months, RM ANOVA was conducted again for the five BRIEF-A outcomes with TIME (pre vs. follow-up) as the within-subject factor and GROUP (NF vs. sham) as the between-subjects factor. Descriptive data is provided for participants who completed the 12-month follow-up measurement (*n* = 10).

#### 2.4.8. Data preparation and interpretation

A winsorizing approach was applied to all data, in which outlying values (i.e., >three SD from the mean) for each group were replaced with a less extreme value (i.e., mean ± three times the SD) to minimize their influence (Sullivan et al., 2021). For the NF data, a total of fifteen missing blocks and four blocks with clearly erroneous values were replaced for individual participants (i.e., end baseline replaced with start baseline from the same session, NF block replaced with previous NF block from the same session, and transfer block replaced with transfer block from previous session).

For statistical tests, a *p*-value of ≤0.05 was used to determine significant differences. Multiple test correction was not applied for the RM ANOVAs due to the clear *a priori* hypotheses about the effects based on previous research. However, the interpretation and discussion of the results took into account the increased risk of type I errors that can occur due to multiple testing (Streiner and Norman, 2011). To correct for multiple comparisons in the exploratory correlational analyses, the Benjamini-Hochberg adjustment with a false discovery rate of 0.05 was applied (Chen et al., 2017). In case of violations of sphericity in RM ANOVA, the Greenhouse-Geisser correction was applied and corrected degrees of freedom and *p*-values were reported. The effect size for RM ANOVA was indicated by partial eta squared ($\eta_{p}^{2}$) and interpreted as small (<0.06), medium (≥0.06), or large (≥0.14). Pearson correlations were interpreted as small (<0.3), medium (≥0.3), or large (≥0.5).

For all analyses of transfer effects, a conservative approach was used (i.e., comparison of NF vs. sham rather than NF responders vs. sham) to determine if the findings from previous research on FM theta NF could be replicated in this subclinical population. Finally, in order to detect a medium effect of $\eta_{p}^{2}$ = 0.06 (i.e., smallest effect size of interest) with 95% power in a within-between subjects RM ANOVA design with eight or nine measurements (two groups, α = 0.05, correlation among repeated measures = 0.5, non-sphericity correction *epsilon* = 0.5), G*Power 3.1.9.4, suggested we needed at least nineteen or seventeen participants per group, respectively. For the same RM ANOVA design with two measurements (two groups, α = 0.05, β = 0.95, $\eta_{p}^{2}$ = 0.06, correlation among repeated measures = 0.7, non-sphericity correction *epsilon* = 1), at least seventeen participants per group are required. Statistical analyses were performed using SPSS 26.

## 3. Results

### 3.1. Group characteristics

Table 2 presents an overview of the demographics, ITP, and questionnaire scores for the NF and sham groups. Age [*t*(56) = 0.689, *p* = 0.494], education level [*X*^2^(2, *n* = 58) = 1.040, *p* = 0.595], gender [*X*^2^(1, *n* = 58) = 0.305, *p* = 0.581], and ITP [*t*(56) = 0.964, *p* = 0.339] did not differ between the NF and sham groups. Similarly, there were no significant differences between the groups in the number of reported depressive symptoms [*t*(54) = −0.212, *p* = 0.833], with both groups scoring on average in the minimal range (≤13). Regarding ADHD symptoms, both groups had a similar number of attentional symptoms [*t*(54) = 0.287, *p* = 0.776]. In the NF group, 69.0% of participants reported four or more attentional symptoms, and in the sham group 62.1%. The NF and sham groups also did not differ regarding the number of hyperactivity symptoms [*t*(54) = −1.103, *p* = 0.275], 31.0% of participants in the NF group and 48.3% in the sham group reported four or more hyperactivity symptoms.

**TABLE 2:** Demographic characteristics and questionnaire scores for the NF and sham groups.

	NF group (*n* = 29)	Sham group (*n* = 29)
	***n* (%)**	***n* (%)**
Education level (low / intermediate / high)	1 (3.4%) / 12 (41.4%) / 16 (55.2%)	0 (0%) / 13 (44.8%) / 16 (55.2%)
Sex (female)	18 (62.1%)	20 (69.0%)
	**M (SD)**	**M (SD)**
Age (in years)	34.5 (11.8) Range: 20–60	32.5 (9.8) Range: 20–52
ITP	6.1 (0.8)	5.9 (0.9)
BDI-II total score at T2	9.4 (7.4)^1^	9.8 (6.2)
ZVAH attentional symptoms at T2	5.1 (2.6)^1^	4.9 (3.0)
ZVAH hyperactivity symptoms at T2	2.8 (2.1)^1^	3.6 (3.0)

ITP, individual theta peak; BDI-II, Beck Depression Inventory II; ZVAH, Self-report questionnaire on attention problems and hyperactivity for adult- and childhood (adult version).

^1^Information is missing for two participants (*n* = 27).

In the NF group, nine participants reported receiving a diagnosis of attention deficit disorder (ADD). Additionally, three participants had been diagnosed with ADHD, four with autism spectrum disorder (with one also reporting bipolar disorder), and one with post-traumatic stress disorder. The sham group included nine participants reporting a diagnosis of ADD, seven with ADHD (one of which additionally reported borderline personality disorder), two with autism spectrum disorder, and one reported a history of depression and anorexia. For most participants, the diagnosis was confirmed by a mental healthcare organization through their general practitioner, but for three participants, the reported diagnosis was either not confirmed or no permission was obtained from the participant. In the NF group and sham group, there were three and four participants, respectively, who suspected to suffer from AD(H)D, but this was not confirmed by a medical expert (yet).

During the NF training, nine participants in the NF group reported taking medications that could potentially impact their cognition. These medications included seven stimulants, one antidepressant, and one atypical antipsychotics. In the sham group, eleven participants were taking medications, including eight stimulants (one combined with an antidepressant), two antidepressants, and one an antidepressant plus an anticonvulsant. Two participants in the sham group voluntarily ceased taking stimulants until after the post-measurement was conducted.

### 3.2. NF training effects

#### 3.2.1. NF training effects in the full groups

**FIGURE 4:**
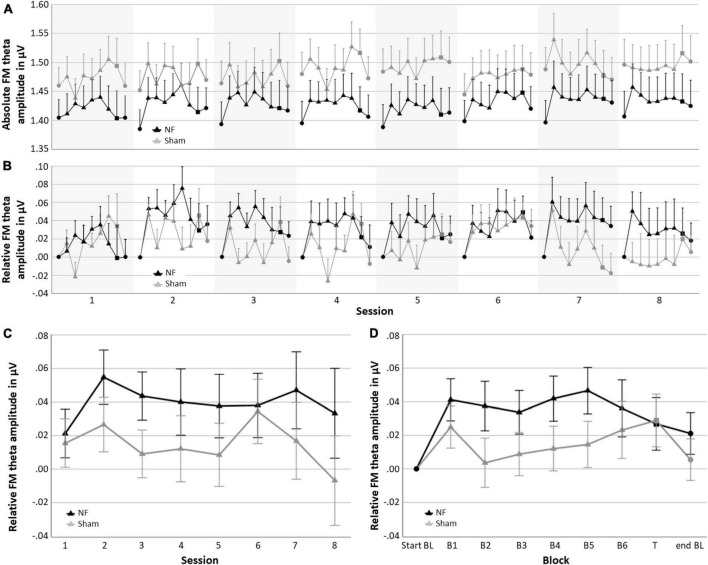
Neurofeedback (NF) results for the NF group and sham group; **(A)** mean absolute FM theta amplitude for each block per session, **(B)** mean FM theta amplitude relative to the respective start baseline for each block per session, **(C)** learning Index 1: Mean FM theta amplitude relative to the respective start baseline per session across the six NF blocks (i.e., session-to-session changes), and **(D)** learning Index 2: Mean FM theta amplitude relative to the respective start baseline per block across sessions (i.e., dynamical changes within sessions). Error bars indicate the standard error of the mean. • = baseline (BL) start or end,▲ = NF block(s), and ■ = transfer (T) block.

Figure 4 gives an overview of the absolute FM theta amplitude (A) and FM theta amplitude relative to the respective baseline (B) for all blocks and sessions for both the NF and sham group, as well as the session-to-session changes in the NF blocks (C) and the dynamical changes within sessions for each block (D). The descriptive Figures 4A, B show that visually the absolute FM theta amplitudes seem higher in the sham group relative to the NF group, however, this observed trend is reversed when FM theta amplitudes are considered relative to the respective baselines. Additionally, Table 3 gives an overview of the depicted estimated marginal means with their 95% confidence interval.

**TABLE 3:** Estimated marginal means for the NF sessions in Learning Index 1 and the blocks in Learning Index 2 for the NF group and sham group.

		NF group				Sham group			
				**95% CI**				**95% CI**	
	**Session**	***M***	***SEM***	**Left**	**Right**	***M***	***SEM***	**Left**	**Right**
Learning Index 1	1	0.021	0.014	−0.008	0.05	0.016	0.014	−0.013	0.045
	2	0.055	0.016	0.022	0.087	0.027	0.016	−0.006	0.059
	3	0.044	0.014	0.015	0.072	0.009	0.014	−0.02	0.038
	4	0.04	0.02	0	0.08	0.012	0.02	−0.027	0.052
	5	0.038	0.019	0	0.075	0.009	0.019	−0.029	0.046
	6	0.038	0.019	0	0.076	0.034	0.019	−0.004	0.073
	7	0.047	0.023	0.001	0.093	0.017	0.023	−0.029	0.063
	8	0.033	0.027	−0.02	0.087	−0.007	0.027	−0.06	0.047
	**Block**	***M***	***SEM***	**Left**	**Right**	***M***	***SEM***	**Left**	**Right**
Learning Index 2	B1	0.041	0.013	0.016	0.066	0.025	0.013	0	0.05
	B2	0.037	0.015	0.008	0.067	0.004	0.015	−0.026	0.033
	B3	0.034	0.013	0.008	0.06	0.009	0.013	−0.017	0.035
	B4	0.042	0.013	0.015	0.069	0.012	0.013	−0.015	0.039
	B5	0.047	0.014	0.019	0.074	0.015	0.014	−0.013	0.042
	B6	0.036	0.017	0.002	0.07	0.023	0.017	−0.011	0.057
	T	0.027	0.016	−0.004	0.058	0.029	0.016	−0.002	0.06
	End BL	0.021	0.012	−0.004	0.046	0.006	0.012	−0.019	0.03

The start baseline is not included because this is zero. SEM, standard error of the mean; CI, confidence interval; B1, neurofeedback block 1, etc. T, transfer block; End BL, end baseline (resting state EEG).

When statistically assessing relative changes in amplitude from session-to-session (i.e., Learning Index 1), a RM ANOVA for FM theta revealed no significant interaction effect SESSION × GROUP [*F*_(4.038,226.119)_ = 0.364, *p* = 0.836], and no main effect of GROUP [*F*_(1,56)_ = 1.815, *p* = 0.183], which both is contrary to expectations. Additionally, no main effect was found for SESSION [*F*_(4.038,226.119)_ = 0.692, *p* = 0.559], and also not for delta, alpha, and beta amplitudes.

Regarding the dynamical changes in amplitude within sessions (i.e., Learning Index 2), a RM ANOVA for FM theta again showed no significant interaction effect BLOCK × GROUP [*F*_(3.069,171.880)_ = 1.124, *p* = 0.342] or main effect for GROUP [*F*_(1,56)_ = 1.512, *p* = 0.224]. There was, however, a main effect for BLOCK [*F*_(3.069,171.880)_ = 2.818, *p* = 0.039, $\eta_{p}^{2}$ = 0.048], see Figure 4D. For delta, alpha, and beta, there were also significant main effects for BLOCK [delta: *F*_(3.433,192.230)_ = 21.575, *p* < 0.001, $\eta_{p}^{2}$ = 0.278; alpha: *F*_(2.431,136.127)_ = 12.299, *p* < 0.001, $\eta_{p}^{2}$ = 0.180; and beta: *F*_(2.366,132.476)_ = 6.500, *p* = 0.001, $\eta_{p}^{2}$ = 0.104]. In both groups, delta decreased in amplitude across blocks within sessions, while alpha and beta showed an increase. A full overview of all RM ANOVA results can be found in Supplementary Appendix Tables 1, 2.

#### 3.2.2. NF training effects responders in the NF groups vs. sham group

**FIGURE 5:**
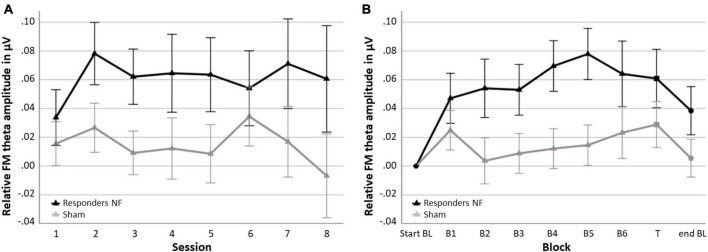
Neurofeedback (NF) Learning indices for the responders in the NF group and the sham group. **(A)** Learning Index 1: Mean FM theta amplitude relative to the respective start baseline per session across the six NF blocks (i.e., session-to-session changes) and **(B)** Learning Index 2: Mean FM theta amplitude relative to the respective start baseline per block across sessions (i.e., dynamical changes within sessions). Error bars indicate the standard error of the mean. • = baseline (BL) start or end, ▲ = NF block(s), and ■ = transfer (T) block.

In the NF group, 62.1% of the participants were classified as responders (*n* = 18) and 37.9% as non-responders (*n* = 11). In the sham group 51.7% (*n* = 15) were also classified as responders and 48.3% (*n* = 14) as non-responder. Figure 5 shows the session-to-session changes in FM theta amplitude in the NF blocks and the dynamical changes within sessions for each block for the responders in the NF group and the sham group. The RM ANOVA for session-to-session changes (i.e., Learning Index 1) revealed a significant main effect of GROUP [*F*_(1,45)_ = 4.269, *p* = 0.045, $\eta_{p}^{2}$ = 0.087]. Responders in the NF group exhibited significantly higher FM theta amplitudes throughout the eight NF sessions, starting from the first NF session, in comparison to the sham group. However, the study revealed a lack of significant interaction effect between SESSION × GROUP [*F*_(3.874,174.319)_ = 0.432, *p* = 0.779]. Both results together suggest that there was a consistent difference in FM theta upregulation starting from the first session, without a further significant increase throughout the NF session among the NF responders compared to the sham group. Furthermore, there was no significant main effect for SESSION [*F*_(3.874,174.319)_ = 0.467, *p* = 0.754].

Repeated measures ANOVA for dynamical changes in FM theta amplitude within sessions (i.e., Learning Index 2) revealed similar results. Again, there was a significant main effect of GROUP [*F*_(1,45)_ = 4.692, *p* = 036, $\eta_{p}^{2}$ = 0.094]; across the blocks, responders in the NF group showed a higher FM theta amplitude compared to the sham group, including the end baseline block, in which participants were not asked to upregulate their FM theta. There was no other significant interaction effect of BLOCK × GROUP [*F*_(2.907,130.834)_ = 1.612, *p* = 0.191], suggesting that the difference between the responders in the NF group and the sham group on FM theta was consistently higher across blocks. Finally, a significant main effect of BLOCK was found [*F*_(2.907,130.834)_ = 3.755, *p* = 0.013, $\eta {}_{p}^{2}$ = 0.077].

#### 3.2.3. Characteristics responders vs. non-responders

Table 4 provides an overview of demographic characteristics, questionnaire scores, and NF outcomes for the responders and non-responders in the NF group. Overall, the two groups were very similar, but the responders included relatively more participants without a psychiatric disorder [*X*^2^(2, *n* = 29) = 7.735, *p* = 0.021].

**TABLE 4:** Demographic characteristics and questionnaire scores, and NF outcomes of the responders and non-responders in the NF group.

	Responders (*n* = 18)	Non-responders (*n* = 11)
	**M (SD)**	**M (SD)**
Mean FM theta amplitude during first resting state in NF session 1	1.425 (0.140)	1.372 (0.115)
Motivation	5.7 (1.0)	5.6 (1.0)
Commitment	5.2 (1.2)	5.7 (1.1)
Difficulty	4.5 (0.7)	4.8 (0.7)
Age	34.1 (11.1)	35.0 (13.5)
BRIEF-A total score (T1)	155.1 (18.7)	154.9 (18.8)
BRIEF-A working memory (T1)	18.6 (2.6)	19.7 (3.0)
BRIEF-A shift (T1)	13.1 (3.3)	13.6 (3.0)
BRIEF-A task monitor (T1)	14.9 (2.2)	14.0 (2.6)
BRIEF-A inhibit (T1)	16.0 (3.3)	15.5 (3.6)
BDI-II total score (T1)	9.3 (8.2)^1^	9.6 (6.0)^1^
ZVAH attentional symptoms (T2)	4.8 (2.5)^1^	5.7 (2.7)^1^
ZVAH Hyperactivity symptoms (T2)	2.8 (2.1)^1^	2.7 (2.3)^1^
	*n* (%)	*n* (%)
Educational level	Low: 0 (0.0%) Intermediate: 6 (33.3%) High: 12 (66.7%)	Low: 1 (9.1%) Intermediate: 6 (54.5%) High: 4 (36.4.3%)
Sex (female)	11 (61.1%)	7 (63.6%)
Presence of disorder	No diagnosis: 7 (38.9%) ADD : 7 (38.9%) ADHD: 1 (5.6%) ASD: 2 (11.0%) PTSD: 1 (5.6%)	No diagnosis: 2 (18.2%) Suspect AD(H)D: 3 (27.2%) ADD: 2 (18.2%) ADHD: 2 (18.2%) ASD: 1 (9.1%) ASD + bipolar disorder: 1 (9.1%)
Medication use	No medication: 13 (72.2%) Stimulants: 4 (22.2%) Atypical antipsychotic: 1 (5.6%)	No medication: 7 (63.6%) Stimulants: 3 (27.3%) Antidepressant: 1 (9.1%)

BRIEF-A, Behavior Rating Inventory of Executive Function - Adult Version; BDI-II, Beck Depression Inventory II; ZVAH, Self-report questionnaire on attention problems and hyperactivity for adult and childhood (adult version); ADD, attention deficit disorder; ADHD, attention deficit hyperactivity disorder; ASD, autism spectrum disorder; PTSD, post-traumatic stress disorder; T1, pre-measurement; T2, post-measurement.

^1^In both groups, information is missing for one participant. Self-reported motivation, commitment, and difficulty are rated on a seven-point scale and are averaged across eight sessions.

### 3.3. Credibility sham group

A chi-square test of independence showed that there was no significant association between actual and guessed group membership [*X*^2^(1, *n* = 41) = 1.90, *p* = 0.168]. Additionally, there were no significant differences between groups in motivation, commitment, and perceived difficulty during the training. However, for motivation there was a significant main effect of SESSION [*F*_(5.567,311.769)_ = 8.313, *p* < 0.001, $\eta_{p}^{2}$ = 0.129]; both groups showed a decrease in motivation over the course of the sessions. The levels of commitment and perceived difficulty were stable throughout the training. A full overview of the results can be found in Supplementary Appendix Table 3.

Finally, Table 5 provides an overview of demographic characteristics, questionnaire scores, and NF success for participants who completed the follow-up measurement (*n* = 41) and those who dropped out (*n* = 17). Overall, the two groups are very similar, but the group that completed the follow-up appears to have relatively more participants with a disorder.

**TABLE 5:** Demographic characteristics and questionnaire scores, and NF outcomes of participants who completed the follow-up measurement (either 6 or 12 months) and those who dropped out after the post-measurement.

	Completed follow-up (*n* = 41)	Drop-outs (*n* = 17)
	**M (SD)**	**M (SD)**
Motivation	5.7 (0.9)	5.4 (1.0)
Commitment	5.7 (1.0)	5.1 (1.3)
Difficulty	4.6 (0.8)	4.7 (0.9)
Age	33.4 (10.7)	33.6 (11.5)
BRIEF-A total score (T1)	156.4 (18.4)	154.1 (17.9)
BRIEF-A working memory (T1)	19.8 (2.6)	18.9 (2.7)
BRIEF-A shift (T1)	13.3 (2.6)	13.0 (2.6)
BRIEF-A task monitor (T1)	14.9 (2.3)	14.5 (2.0)
BRIEF-A inhibit (T1)	16.0 (3.4)	16.3 (3.3)
BDI-II total score (T2)	9.5 (7.2)	9.9 (5.7)**^1^**
ZVAH attentional symptoms (T2)	5.0 (3.0)	5.0 (2.2)^1^
ZVAH Hyperactivity symptoms (T2)	3.1 (2.7)	3.5 (2.4)^1^
	*n* (%)	*n* (%)
NF success (responder)	33 (80.5%)	14 (82.4%)
Educational level	Low: 0 (0.0%) Intermediate: 16 (39.0%) High: 25 (61.0%)	Low: 1 (5.9%) Intermediate: 7 (41.2%) High: 9 (52.9%)
Sex (female)	26 (63.4%)	12 (70.6%)
Presence of disorder	No diagnosis: 8 (19.5%) Suspect AD(H)D: 5 (12.2%) ADD: 13 (31.8%) ADHD: 6 (14.7%) ADHD + BPD: 1 (2.4%) ASD: 5 (12.2%) ASD + bipolar disorder: 1 (2.4%) PTSD: 1 (2.4%) History of depression + anorexia: 1 (2.4%)	No diagnosis: 7 (41.2%) Suspect AD(H)D: 2 (11.8%) ADD: 5 (29.4%) ADHD: 3 (17.6%)
Medication use during NF training	No medication 26 (63.4%) Stimulants: 10 (24.4%) Antidepressant: 2 (4.9%) Stimulant + anticonvulsant: 1 (2.4%) Stimulant + antidepressant 1: (2.4%) Atypical antipsychotic: 1 (2.4%)	No medication: 12 (70.6%) Stimulants: 4 (23.5%) Antidepressant: 1 (5.9%)

BRIEF-A, Behavior Rating Inventory of Executive Function - Adult Version; BDI-II, Beck Depression Inventory II; ZVAH, self-report questionnaire on attention problems and hyperactivity for adult and childhood (adult version); ADD, attention deficit disorder; ADHD, attention deficit hyperactivity disorder; BPD, borderline personality disorder; ASD; autism spectrum disorder; PTSD, post-traumatic stress disorder; T1, pre-measurement; T2, post-measurement.

^1^Information is missing for two participants (*n* = 15). Self-reported motivation, commitment, and difficulty are rated on a seven-point scale and are averaged across eight sessions.

### 3.4. Transfer effects on behavior

#### 3.4.1. Immediate behavioral transfer effects

**FIGURE 6:**
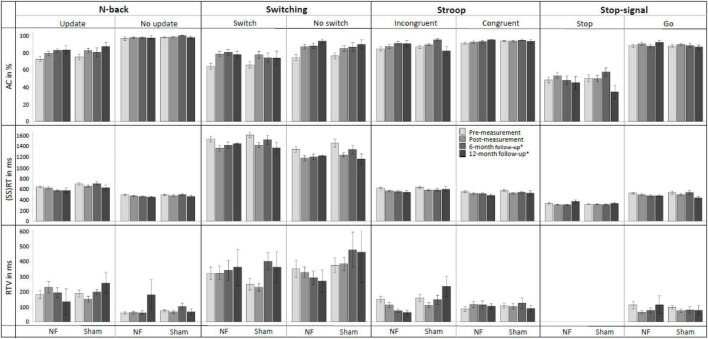
Mean accuracy (AC), reaction time (RT), and RT variability (RTV) for all conditions of the N-back, Switching, Stroop, and Stop-signal task at pre-measurement, post-measurement, 6-month follow-up*, and 12-month follow-up* for the NF group and sham group. Error bars indicate the standard error of the mean. *Due to COVID-19 regulations, not all participants could perform the 6-month follow-up measurement. Therefore, nine participants did a 12-month follow-up measurement instead.

Figure 6 provides an overview of behavioral performance on the four EF tasks (i.e., N-back, Switching, Stroop, and Stop-signal task) at pre-, post-, and follow-up measurements. For the Update condition of the N-back task, RM ANOVAs demonstrated, contrary to our expectations, no significant interaction effects of TIME × GROUP. There was a significant main effect of TIME for AC [*F*_(1,56)_ = 22.148, *p* < 0.001, $\eta_{p}^{2}$ = 0.283] and RT [*F*_(1,56)_ = 7.627, *p* = 0.008, $\eta_{p}^{2}$ = 0.120]; both groups improved their AC and reduced RT immediately after the NF training. There were no significant main effects of GROUP.

For the Switch condition of the Switching task, again contrary to expectations, there were no significant interaction effects TIME × GROUP. For RTV, there was a significant main effect of GROUP [*F*_(1,56)_ = 4.524, *p* = 0.038, $\eta_{p}^{2}$ = 0.075]; the NF group scored significantly higher than the sham group across both measurements. Additionally, there were significant main effects of TIME for AC [*F*_(1,56)_ = 41.460, *p* < 0.001, $\eta_{p}^{2}$ = 0.425] and RT [*F*_(1,56)_ = 36.683, *p* < 0.001, $\eta_{p}^{2}$ = 0.396], both groups improved their AC and reduced RT immediately after the NF training.

For the Incongruent condition of the Stroop task, no significant interaction effects TIME × GROUP or main effects of GROUP were found. There was a significant main effect of time for AC [*F*_(1,56)_ = 7.113, *p* = 0.010, $\eta_{p}^{2}$ = 0.113], RT [*F*_(1,56)_ = 28.787, *p* < 0.001, $\eta_{p}^{2}$ = 0.340], and RTV [*F*_(1,56)_ = 4.976, *p* = 0.030, $\eta_{p}^{2}$ = 0.082]; both groups improved AC and reduced RT and RTV immediately after the NF training.

Finally, for the Stop condition of the Stop-signal task, there was no significant interaction between TIME × GROUP, and again only a significant main effect of TIME for the SSRT [*F*_(1,56)_ = 3.996, *p* = 0.050, $\eta_{p}^{2}$ = 0.067]; both groups improved their SSRT. A full overview of all RM ANOVA results can be found in Supplementary Appendix Table 4.

#### 3.4.2. Long-term behavioral transfer effects

The assessment of NF training effects after 6 months (i.e., pre- vs. 6-month follow-up measurement) revealed significant results for the Update condition of the N-back task. For RT, the RM ANOVA showed as expected a significant interaction effect TIME × GROUP [*F*_(1,30)_ = 4.410, *p* = 0.044, $\eta_{p}^{2}$ = 0.128] and a main effect of GROUP [*F*_(1,30)_ = 6.991, *p* = 0.013, $\eta_{p}^{2}$ = 0.189]. The results showed that both prior to the NF training and 6 months later, the NF group had faster RTs compared to the sham group. However, the difference between groups was even larger at the 6-month follow-up measurement, suggesting specific FM theta NF effects on RT. Additionally, there was a significant main effect of TIME for AC [*F*_(1,30)_ = 6.808, *p* = 0.014, $\eta_{p}^{2}$ = 0.185]; both groups showed higher AC 6 months after the NF training compared to before the training.

For the Switch condition of the Switching task, there was no significant interaction TIME × GROUP. Only a significant main effect of TIME was found for AC [*F*_(1,30)_ = 25.136, *p* < 0.001, $\eta_{p}^{2}$ = 0.456], with both groups showing a higher AC at the 6-month follow-up measurement than before the training.

Regarding the Incongruent condition of the Stroop task, RM ANOVA revealed a significant interaction effect TIME × GROUP [*F*_(1,30)_ = 4.446, *p* = 0.043, $\eta_{p}^{2}$ = 0.129], as well as a main effect of TIME [*F*_(1,30)_ = 7.308, *p* = 0.011, $\eta_{p}^{2}$ = 0.196] for RTV. These results indicate that both groups showed a reduction in reaction time variability (RTV) 6 months after the training, however, the reduction in RTV was greater for the NF group compared to the sham group, suggesting specific FM theta NF effects on RTV. Additionally, significant main effects of TIME were observed for AC [*F*_(1,30)_ = 11.835, *p* = 0.002, $\eta_{p}^{2}$ = 0.283] and RT [*F*_(1,30)_ = 15.931, *p* < 0.001, $\eta_{p}^{2}$ = 0.347]; both groups improved AC and reduced RT 6 months after the NF training.

Finally, considering the Stop condition of the Stop-signal task, no significant interaction effects or main effects were found. A full overview of all RM ANOVA results can be found in Supplementary Appendix Table 5.

### 3.5. Correlations between self-regulation of FM theta and behavioral changes in EFs

Table 6 shows an overview of the Pearson correlations between upregulation success in the NF training and change in behavior (i.e., AC, RT, and RTV) immediately after the NF training (i.e., pre- vs. post-measurement) and in the long-term (i.e., pre- vs. follow-up measurement). The results show that there were no significant associations (i.e., *p* > Benjamini-Hochberg critical value) when considering the whole sample.

**TABLE 6:** Pearson correlations between FM theta upregulation success and the behavioral outcomes accuracy (AC), reaction time (RT), and RT variability (RTV) immediately after training and in the long-term.

		Pre- vs. post-measurement		Pre- vs. 6-month follow-up measurement	
		**(*n* = 58)**		**(*n* = 32)**	
**Task condition**	**Change in**	***r***	***p***	***r***	***p***
Update	AC	−0.067	0.617	−0.171	0.349
	RT	−0.152	0.256	−0.207	0.257
	RTV	−0.024	0.86	0.032	0.86
Switch	AC	−0.177	0.185	−0.337	0.059
	RT	−0.148	0.268	0.069	0.709
	RTV	−0.17	0.203	−0.161	0.379
Incongruent	AC	−0.041	0.758	−0.168	0.357
	RT	0.247	0.061	0.148	0.42
	RTV	0.221	0.095	0.297	0.099
Stop	SSRT	0.085	0.523	0.109	0.553

### 3.6. Transfer effects on FM theta

#### 3.6.1. Immediate transfer effects to FM theta during EF tasks

**FIGURE 7:**
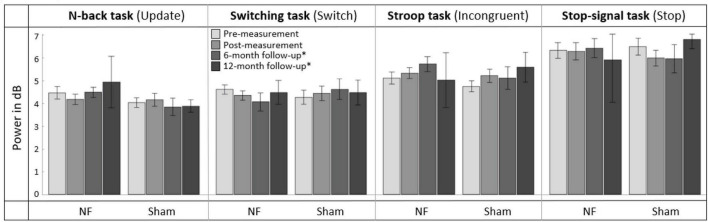
Mean frontal-midline (FM) theta power (±1 Hz and ± 50 ms around the highest peak in FM theta power identified within 100 to 500 ms after stimulus onset between 4 and 8 Hz) for the task conditions requiring EFs (i.e., Update condition of the N-back task, Switch condition of the Switching task, Incongruent condition of the Stroop task, and Stop condition of the Stop-signal task) at pre-measurement, post-measurement, 6-month follow-up*, and 12-month follow-up* for the NF group and sham group. Error bars indicate the standard error of the mean. *Due to COVID-19 regulations, not all participants could perform the 6-month follow-up measurement. Therefore, nine participants did a 12-month follow-up measurement instead.

Figure 7 gives an overview of the mean FM theta power across the pre-, post-, and follow-up measurements. RM ANOVAs of the immediate effects of the NF training on theta power 100 to 500 ms after stimulus onset (i.e., pre- vs. post-measurement), revealed no significant interaction effect TIME × GROUP or main effects for TIME or GROUP for the four task conditions. A full overview of all RM ANOVA results can be found in Supplementary Appendix Table 6.

#### 3.6.2. Long-term transfer effects to FM theta during EF tasks

For the effects after 6 months (i.e., pre- vs. 6-month follow-up measurement), RM ANOVA showed no significant interaction effect TIME × GROUP or main effect for GROUP. There was, however, a significant main effect of TIME for the Incongruent condition of the Stroop task [*F*_(1,28)_ = 7.003, *p* = 0.013, $\eta_{p}^{2}$ = 0.200]; theta power was significantly higher in both groups 6 months after the NF training compared to before the training. A full overview of all RM ANOVA results can be found in Supplementary Appendix Table 7.

### 3.7. Transfer effects to daily life

#### 3.7.1. Immediate transfer effects to EFs in daily life

**FIGURE 8:**
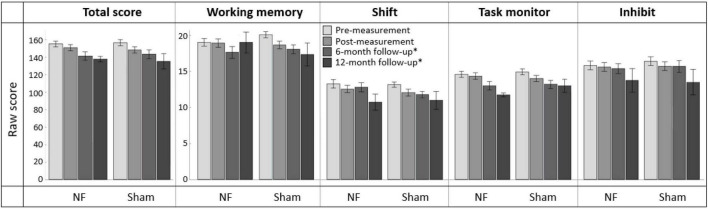
Mean scores on the BRIEF-A total score and subscales Working memory, Shift, Task monitor, and Inhibit at pre-measurement, post-measurement, 6-month follow-up*, and 12-month follow-up* for the NF group and sham group. Error bars indicate the standard error of the mean. *Due to COVID-19 regulations, not all participants could complete the BRIEF-A at 6-month follow-up. Therefore, ten participants completed it at 12-month follow-up.

Figure 8 provides an overview of scores on the BRIEF-A outcomes at pre-, post-, and follow-up measurements. Regarding the immediate effects after the NF training (i.e., pre- vs. post-measurement), RM ANOVA showed a significant interaction effect TIME × GROUP [*F*_(1,56)_ = 5.865, *p* = 0.019, $\eta_{p}^{2}$ = 0.095] and main effect of TIME [*F*_(1,56)_ = 7.863, *p* = 0.007, $\eta_{p}^{2}$ = 0.123] for the Working memory subscale. Contrary to expectation, the sham group showed a larger decrease in complaints than the NF group. Additionally, there were significant main effect of TIME for the BRIEF-A total score [*F*_(1,56)_ = 19.497, *p* < 0.001, $\eta_{p}^{2}$ = 0.258] and subscales Shift [*F*_(1,56)_ = 10.915, *p* = 0.002, $\eta_{p}^{2}$ = 0.163] and Task monitor [*F*_(1,56)_ = 7.287, *p* = 0.009, $\eta_{p}^{2}$ = 0.115]. Both groups showed a decrease in the number of complaints on these BRIEF-A outcomes immediately after the NF training. For the Inhibit subscale, no significant interaction or main effects were found. A full overview of all RM ANOVA results can be found in Supplementary Appendix Table 8.

#### 3.7.2. Long-term transfer effects to EFs in daily life

In total, 39 participants completed the BRIEF-A 6 months after the NF training, and ten participants completed it after 12 months. Regarding the effects after 6 months (i.e., pre- vs. 6-month follow-up measurement), no significant interaction effect TIME × GROUP or main effect for GROUP were found. However, there were significant main effects of TIME for all BRIEF-A outcomes: total score [*F*_(1,37)_ = 48.168, *p* < 0.001, $\eta_{p}^{2}$ = 0.566] and subscales Working memory [*F*_(1,37)_ = 23.317, *p* < 0.001, $\eta_{p}^{2}$ = 0.387], Shift [*F*_(1,37)_ = 12.483, *p* = 0.001, $\eta_{p}^{2}$ = 0.252], Task monitor [*F*_(1,37)_ = 43.887, *p* < 0.001, $\eta_{p}^{2}$ = 0.543], and Inhibit [*F*_(1,37)_ = 10.081, *p* = 0.003, $\eta_{p}^{2}$ = 0.214]. In both groups, the number of complaints measured by these outcomes was significantly lower 6 months after training than before training. A full overview of all RM ANOVA results can be found in Supplementary Appendix Table 9.

## 4. Discussion

This study examined for the first time the effects of FM theta NF on EFs in a (sub)clinical population characterized by notable self-reported EF complaints in daily life. Using a pre/post/follow-up design with a sham NF group, the immediate and long-term effects of an eight-session individualized FM theta NF training were assessed in 58 adults aged 20–60 years. First, it was examined whether the NF training resulted in improved upregulation of FM theta using two NF learning indices. Second, the immediate and long-term transfer effects of the NF training were assessed. This included behavioral performance on proactive and reactive EF tasks (assessing working memory updating, set-shifting, conflict monitoring, and response inhibition), FM theta power during these tasks, and subjective EFs in daily life.

The findings indicate that there is only a significant difference in the upregulation of FM theta between the NF group and sham group when participants who did not respond to the NF are excluded from the analysis. Additionally, the study demonstrates that the NF group displays stronger improvement in behavioral performance for working memory updating and conflict monitoring at the 6-month follow-up. However, there are no NF-specific effects identified immediately after the NF training. Furthermore, no immediate or long-term effects specific to the NF training are observed for FM theta power during the EF tasks and EFs in daily life. These results regarding FM theta upregulation and transfer effects, as well as predictors of NF learning and NF training relative to other interventions for EFs, are discussed in more detail below.

### 4.1. Effects of non-responders on training efficacy

Our findings indicate that FM theta amplitudes are not significantly different between the NF and sham groups, when both indices of NF learning are examined and all participants are considered. This result cannot be explained by initial group differences or participants’ awareness of group assignment. Yet, the proportion of participants non-respone in the NF group (i.e., 37.9%) could play a role, which is a rate consistent with the findings of previous NF studies (e.g., Alkoby et al., 2018). Excluding these non-responders, the analysis reveals that the responders in the NF group exhibit the anticipated outcome of higher FM theta amplitudes compared to the sham group. However, an increasing number of NF sessions for the responders in the NF group greater upregulation of FM theta, instead remaining constant when compared to the sham group. These findings differ from previous studies in healthy young and older adults, which demonstrated a greater increase in FM theta in the NF group also across sessions without accounting for non-responders (Wang and Hsieh, 2013; Enriquez-Geppert et al., 2014a; Brandmeyer and Delorme, 2020; Eschmann and Mecklinger, 2022). Inherent to their EF complaints, it is possible that the participants in this study had more difficulty retaining instructions, sitting still, focusing on the feedback, and maintaining the use of strategies and adapting them as needed (e.g., Roth et al., 2005), which might have reduced their ability to learn from the feedback and self-regulate FM theta. Moreover, it may be that our participants had lower baseline levels of FM theta compared to healthy individuals, which is disadvantageous for upregulation success (Weber et al., 2020). Finally, the current study included a highly heterogeneous group of participants in terms of age, educational level, presence of psychiatric disorder, and medication use. This may have resulted in weaker learning compared to previous FM theta NF studies that included mostly healthy, young, highly educated students (e.g., Hooghe et al., 2010). It may be necessary to conduct a greater number of sessions in individuals with (sub)clinical conditions to attain the same level of upregulation observed in a healthy sample. For example, NF for ADHD in clinical settings typically consists of 30–40 sessions (Arns et al., 2020).

It is worth noting that the learning curve for session-to-session changes (reflected in Learning Index 1) in the NF group shows a pattern similar in the first part of the training to that observed in other studies on FM theta NF. Nonetheless, in the second part of the NF training, FM theta upregulation appears weaker in our (sub)clinical group, although participants receive the same number of sessions with similar training intensity. A recommended next step is to conduct a mega-analysis to accurately evaluate the overall learning curve of FM theta NF training. By pooling raw data from all FM theta NF studies, a mega-analysis retains a higher level of detail compared to a meta-analysis which is based on summary statistics (Eisenhauer, 2021).

Another difference from priour studies is that effects are not specific to theta activity (Enriquez-Geppert et al., 2014a; Eschmann and Mecklinger, 2022), but are also found in adjacent frequency bands. Both groups showed a significant decrease in delta activity and an increase in alpha and beta activity within sessions. However, one would expect an opposite pattern of an increase in delta and a decrease in alpha and beta due to a gradually increasing level of drowsiness and mind wandering and reduced effort in applying mental strategies over the course of a session (MacLean et al., 2012; Kam et al., 2022). Our contrary findings may indicate an attempt by the brain to maintain attention and focus on applying mental strategies by increasing mental effort (e.g., Klimesch, 2012; Pershin et al., 2023).

Interestingly, approximately 51.7% of participants in the sham group were also classified as a responder. This suggests that, despite receiving sham feedback, the sham group demonstrated a certain degree of FM theta upregulation compared to the start baseline (see Figure 4D) which is rather in contrast to previous studies with healthy participants (e.g., Enriquez-Geppert et al., 2014b; Eschmann and Mecklinger, 2022). The current findings imply that the utilization of suggested mental strategies alone might have contributed to the enhancement of FM theta. However, it is important to note that the effectiveness of these strategies is further augmented by receiving accurate feedback regarding actual brain activity. Two implications follow: First, future studies should explore the association between specific strategies and the increase of FM theta, providing further insights into the underlying mechanisms. Second, these findings also raise the question of identifying an optimal sham group and stimulating discussions regarding the use of real self-regulation of random neural frequencies as an active control group (e.g. in Eschmann and Mecklinger, 2022). Instead of merely replaying feedback from a matched participant in the experimental groups, the alternative approach could contribute to a more accurate interpretation of the results.

### 4.2. Predictors of NF learning ability

The responders and non-responders in the NF group exhibited similarities regarding demographics, questionnaire scores, and NF training-related factors. However, it is worth noting that among the non-responders there is a higher proportion of participants that report having or suspecting a psychological diagnosis. The presence of specific disorders and disorder-related features may impact the ability to self-regulate specific brain features. For example, fMRI-based NF training targeting anterior cingulate cortex (ACC) activity in schizophrenia patients resulted in activation of the dorsal ACC (better known as MCC Vogt, 2016), whereas healthy controls activated the rostral ACC (Cordes et al., 2015). Patients with schizophrenia may have activated the dorsal subregion as a compensatory mechanism to regulate the ACC signal, given that they commonly experience impairments in the rostral ACC (e.g., Habel et al., 2010). Additionally, comorbid conditions, such as anxiety or sleeping difficulties, might further impact the ability to learn and benefit from NF (e.g., Rasch and Born, 2013; Koush et al., 2017).

An important question in the field of interventions is whether healthy individuals or patients get the most benefit from trainings. This question has important implications for the design and implementation of interventions, because it has the potential to inform how to optimize the impact of NF. One hypothesis assumes that individuals with more pronounced (sub)clinical impairments have theoretically greater capacity for improvement, whereas the Matthew principle states that those who begin with an advantage will accumulate more advantage over time (Rigney, 2010). NF is an active treatment, as opposed to passive treatments such as medication, and may require some self-regulation skills initially (Weber et al., 2020). For instance, a systematic review by Weber et al. (2020) found that higher baseline levels of the trained neural parameter was the strongest predictor for upregulation success. This finding proposes that participants with higher baseline activity have an advantage in improving their self-regulation ability and would therefore benefit more from an NF intervention. However, in the current study, no differences were found between responders and non-responders in terms of baseline levels of FM theta in the first session. In addition to possible neural activity, other psychological or neurophysiological factors may also play a role in NF learning, e.g. strategies that participants use to self-regulate brain activity (Autenrieth et al., 2020) or anatomical differences e.g. regarding the MCC as an FM theta generator (Enriquez-Geppert et al., 2013b). Overall, more research is needed to better understand the underlying mechanisms of non-response to NF, identify predictors of NF learning ability, and explore ways to improve NF responsiveness, for instance by increasing neuroplasticity.

### 4.3. NF-specific transfer to long-term EF behavioral performance

The main result of this study is the behavioral transfer effect from FM theta NF observed at the 6 months follow-up: the NF group demonstrates greater reductions in RT during working memory updating and in RTV during conflict monitoring compared to the sham group. Faster responding after NF training might indicate increased efficiency or engaged processing, while higher consistency in response speed suggests fewer instances of attention lapses (Tamm et al., 2012; Brenner and Smeets, 2018) through self-regulation of FM theta. In contrast, immediately after the NF training, only repetition or other non-specific effects are present, as evidenced by improved behavioral performance in both groups on all EFs. The lack of immediate effects does not meet our expectations and are inconsistent with findings from FM theta NF studies in healthy participants (Wang and Hsieh, 2013; Enriquez-Geppert et al., 2014a; Brandmeyer and Delorme, 2020; Eschmann and Mecklinger, 2022).

Given the later onset of NF effects, the existing literature on NF studies in clinical groups suggests that transfer effects may be more pronounced when studied after a period of time following the intervention, rather than immediately after NF training (e.g., Garcia Pimenta et al., 2021). The time delay in the appearance of transfer effects after NF can be attributed to the time required for neuroplastic changes to manifest fully (Ros et al., 2014). Visual inspection of data from participants who completed the 12-month follow-up measurement supports this idea of increased NF effects with time (see Figure 5). The lack of NF-specific effects immediately after the NF training could also be due to the relatively lower FM theta upregulation in our NF group compared to previous studies on healthy individuals. Immediate behavioral effects might be present if we had excluded non-responders and compared only the responders in the NF group with the sham group. However, we adopted a conservative approach and only tested our *a priori* hypotheses to replicate the previous FM theta NF studies in this (sub)clinical sample.

Regarding the specific transfer profile in this study, long-term behavioral effects of NF are found as expected for proactive aspects, but only for working memory updating and not for set-shifting. Furthermore, the study demonstrates novel findings regarding the impact of FM theta NF on RTV in conflict monitoring, which is a newly included behavioral outcome. Interestingly, this effect was observed in a reactive task rather than a proactive one. It may be that RTV may capture not only the RT variability driven by the reactive conflict processes, but also the variability in RT driven by sustained attention, thus encompassing both reactive and proactive aspects.

Regarding the analysis of long-term effects after 6 months, it is important to note that there was insufficient power to detect medium effects in RM ANOVA. COVID-19 restrictions prevented 30% of the sample from completing the 6-month follow-up measurement, resulting in a sample size of fourteen participants in the sham group instead of the seventeen required based on power calculations. Furthermore, participants who completed the follow-up were relatively more likely to report a psychiatric diagnosis than those who did not participate. It may be that individuals who dropped out experienced fewer EF complaints in their daily lives after 6 or 12 months and therefore did not see a need to participate in the follow-up measurement. This factor is a commonly possible variable in studies that involve multiple measurements. Finally, alterations in medication use during the follow-up period may have confounded the effects.

### 4.4. No NF-specific transfer to FM theta during EF tasks

The results of this study show that there is no immediate impact of NF training on FM theta power during EF task performance. This is consistent with the findings of Brandmeyer and Delorme (2020) and Eschmann and Mecklinger (2022), but contradicts the results from Enriquez-Geppert et al. (2014a). In addition, this study was the first to investigate the long-term effects of FM theta NF on FM theta power during EF tasks, but again no significant effects were found.

The finding of unaffected FM theta power during EFs may be surprising in the context of the long-term behavioral improvements specific to FM theta NF. However, an explanation may be that the NF training in this (sub)clinical group had an impact on neural parameters related to EFs other than FM theta power, such as improved theta connectivity in the higher-order network or frequency coupling (Enriquez-Geppert et al., 2014a). It could be that the need for cognitive control, reflected by an increase in FM theta power (Cavanagh and Frank, 2014), decreases after FM theta NF due to more efficient execution of cognitive control processes over these other neural parameters underlying EFs. Overall, knowledge of the neural mechanisms underlying NF and specifically FM theta NF is still quite limited and further research with brain imaging technique is needed.

### 4.5. No NF-specific transfer to EFs in daily life

The observed improvement in EFs in daily life, immediately and 6 months after NF training and visually also 12 months afterward, observed in both the NF group and sham group, suggests that the effects are not specific to the NF training but rather related to other factors. These may include non-specific factors related to the context of NF training (e.g., learning to sit still and avoiding muscular artifacts), but also more general non-specific factors such as the benefits of cognitive training, psychosocial influences expectation effects, support and praise from a trainer, repetition-related improvements and natural fluctuations such as spontaneous remission (Micoulaud-Franchi and Fovet, 2018; Ros et al., 2020; Garcia Pimenta et al., 2021).

One explanation for the lack of NF-specific improvement in EFs in daily life, despite improved long-term objective performance, may stem from differences in what is assessed by subjective and objective EF measures. Subjective measures assess an individual’s typical performance in a specific time period, usually involving the integration of multiple cognitive functions, which can be influenced by factors such as perceived stress, depressive symptoms, personality, and self-efficacy beliefs (Facal et al., 2020; Smit et al., 2021). For EFs specifically, the subjective assessment is challenging because EFs are abstract and difficult for people to grasp, unlike more concrete cognitive functions like memory, which are easier to understand. Objective tests, on the other hand, provide a snapshot of a specific EF and require optimal performance and motivation, but may lack ecological validity and sensitivity/specificity to subtle impairments due to successful compensation by the participant (Chaytor et al., 2006). Subjective and objective measures are therefore often only weakly related to each other (e.g., Fuermaier et al., 2015). However, the inclusion of subjective measures is important since the ultimate goal of interventions is to achieve clinically relevant improvements.

### 4.6. No correlations between self-regulation of FM theta and behavioral changes in EFs

Our results show no significant association between FM theta increases and EFs tasks immediately and 6 months after NF training, raising several questions. First, beyond the experimental paradigms, what are the key components of real-world behavior that contribute to FM theta upregulation? In addition, this prompts us to investigate how these aspects can be more effectively measured and ultimately correlated with NF learning indices. Cohen (2014) suggests that FM theta is a preferred frequency band of the brain for EFs because many natural behaviors that are monitored and regulated by the brain, such as typing on a keyboard and speaking, involve temporally sequential micro-actions within the theta frequency range. A recent study addressed the issue of measuring FM theta of real-world behaviors by employing a fully immersive virtual-reality navigation task leading to FM theta modulations, which is a translational model of single-unit electrophysiological recordings from freely moving rodents to a task mimicking real-life goal directed behavior (Lin et al., 2022). This study provides an example for future studies of an effective measure to study the relation between FM theta increases during NF and FM theta during more real-world activities.

### 4.7. NF training relative to other interventions for EFs

Currently, there are no widely accepted standardized protocols or guidelines for the treatment of EF impairments. One of the rehabilitation treatments available is Goal Management Training, where patients learn to become aware of their deficits and improve their ability to perform everyday tasks through psychoeducation, narrative examples, mindfulness exercises, and other tasks (Levine et al., 2000). It typically involves 20 h of training and has been shown to produce small to moderate positive outcomes in terms of both (everyday) EF task performance and in patients’ subjective EF ratings, which can be maintained at follow-up assessments (Stamenova and Levine, 2018). Computer-based cognitive training is another form of training that involves the repetitive performance of tasks involving affected functions, for instance working memory training (Melby-Lervåg and Hulme, 2013). There is, however, limited evidence for the specific effectiveness of this treatment type for EF impairments (Van de Ven et al., 2016). In general, most interventions, programs, and approaches for improving EFs produce immediate specific effects that do not transfer to other domains or daily life (Diamond and Ling, 2016).

Frontal-midline theta NF has the potential to broaden the clinical options for treating EF impairments by utilizing a neuroscientific approach, presenting a new avenue for improvement. In general, it is proposed that an intervention will result in transfer if the intervention and the transfer task involve overlapping processing mechanisms and recruit similar brain regions (e.g., Dahlin et al., 2008). Moreover, the intervention must specifically target and modify the shared underlying processing mechanisms to lead to task transfer (Lövdén et al., 2010), which may explain why most of the EF interventions mentioned above do not have transfer effects. In contrast, FM theta NF has the ability to directly increase the upregulation of FM theta, thus modifying the shared underlying processing mechanisms of EFs (e.g., Enriquez-Geppert et al., 2014a). However, our results suggest, in a (sub)clinical sample this applies only to responders and not to the entire NF group. Furthermore, it should be noted that NF is not the only way to target FM theta. For example, Anguera et al. (2013) found that video game training was also able to increase FM theta power in older adults (60–85 years old) compared to both an active and passive control group. Training led also to improvements in multitasking, sustained attention, and working memory, with some effects lasting for up to 6 months.

Ideally, a treatment protocol for EF impairments, whether it is a single treatment or a combination of treatments, should be tailored to the individual subject to achieve maximum benefit and should be customized based on factors such as severity of EF impairments, presence of other cognitive impairments, general functioning, and personal preferences of the subject. In addition, for NF, personalized protocols that are based on the subject’s characteristics can be used to optimize NF learning (Alkoby et al., 2018). Future studies should aim to identify predictors of effectiveness of different types of EF interventions and explore strategies for treatment stratification.

## Data availability statement

The raw data supporting the conclusions of this article will be made available by the authors, without undue reservation.

## Ethics statement

The studies involving human participants were reviewed and approved by the Ethic Committee of the Faculty for Social Sciences, University of Groningen, Netherlands. The patients/participants provided their written informed consent to participate in this study.

## Author contributions

DS: conceptualization, project administration, data curating, analysis, visualization, and writing-original draft. CD: analysis, visualization, and reviewing. JK and OT: conceptualization, supervision, and reviewing. RH: software, software set up, and reviewing. SE-G: conceptualization, methodology, supervision, writing—original draft, and review and editing. All authors contributed to the article and approved the submitted version.
