# Supplementary material for: Long-term improvements in executive functions after frontal-midline theta neurofeedback in a (sub)clinical group
Source: Front Hum Neurosci. 2023 Jun 9;17:1163380. doi: 10.3389/fnhum.2023.1163380 (PMC10290172; doi:10.3389/fnhum.2023.1163380)
Supplement: Supplementary file 1 [file Data_Sheet_1.PDF]

Table 1: *Repeated measures ANOVA results for session-to-session changes in amplitude (Learning index 1) per frequency.*

**Frequency**	**Factor**	**Source**	**Sum of Squares**	***df***	***F***	***p***	η^2_p
Theta	Within-subject	Session	.053	3.707	.623	.635	.011
(ITP ± 1)		Session * Group	.031	3.707	.369	.816	.007
		Error	4.758	207.572			
	Between-subject	Group	.199	1	2.292	.136	.039
		Error	4.871	56			
Delta	Within-subject	Session	.110	3.919	.773	541	.014
(ITP - 3.5-1.5 Hz)		Session * Group	.066	3.919	.465	.758	.008
		Error	7.947	219.460			
	Between-subject	Group	.309	1	2.163	.147	.037
		Error	7.999	56			
Alpha	Within-subject	Session	.055	5.499	1.377	.228	.024
(ITP + 3-5 Hz)		Session * Group	.060	5.499	1.489	.187	.026
		Error	2.248	307.959			
	Between-subject	Group	.103	1	2.283	.136	.039
		Error	2.526	56			
Beta	Within-subject	Session	.002	3.982	.362	.835	.006
(ITP + 7-24 Hz)		Session * Group	.001	3.982	.243	.913	.004
		Error	.341	223.019			
	Between-subject	Group	.029	1	4.465	.039*	.074
		Error	.363	56			

*Note: ITP = individual theta peak. Significant * p ≤ .05.*

Table 2: *Repeated measures ANOVA results for dynamical changes in amplitude within sessions (Learning index 2) per frequency.*

**Frequency**	**Factor**	**Source**	**Sum of Squares**	***df***	***F***	***p***	η^2_p
Theta	Within-subject	Block	.026	2.987	.770	.512	.014
(ITP ± 1 Hz)		Block * Group	.047	2.987	1.427	.237	.025
		Error	1.858	167.285			
	Between-subject	Group	.119	1	1.958	.167	.034
		Error	3.402	56			
Delta	Within-subject	Block	.730	2.914	17.793	< .001*	.241
(ITP - 3.5-1.5 Hz)		Block * Group	.060	2.914	1.468	.226	.026
		Error	2.297	163.195			
	Between-subject	Group	.254	1	2.417	.126	.041
		Error	5.886	56			
Alpha	Within-subject	Block	.260	2.712	9.648	< .001*	.147
(ITP + 3-5 Hz)		Block * Group	.030	2.712	1.114	.342	.020
		Error	1.508	151.871			
	Between-subject	Group	.049	1	1.172	.284	.020
		Error	2.319	56			
Beta	Within-subject	Block	.013	2.758	7.382	< .001*	.116
(ITP + 7-24 Hz)		Block * Group	.006	2.758	3.291	.026*	.056
		Error	.097	154.459			
	Between-subject	Group	.017	1	3.531	.065	.059
		Error	.268	56			

*Note: ITP = individual theta peak. Significant * p ≤ .05.*

Table 3: *Repeated measures ANOVA results for motivation, commitment, and perceived difficulty across sessions.*

**Outcome**	**Factor**	**Source**	**Sum of Squares**	***df***	***F***	***p***	η_p^2
Motivation	Within-subject	Session	32.052	5.567	8.313	< .001*	.129
		Session * Group	5.784	5.567	1.500	.183	.026
		Error	215.914	311.769			
	Between-subject	Group	1.690	1	.229	.634	.004
		Error	412.948	56			
Commitment	Within-subject	Session	1.612	5.022	.953	.447	.017
		Session * Group	.896	5.022	.530	.754	.009
		Error	94.711	281.240			
	Between-subject	Group	6.401	1	.613	.437	.011
		Error	584.737	56			
Difficulty	Within-subject	Session	12.457	7	1.162	.324	.020
		Session * Group	9.110	7	.849	.547	.015
		Error	600.558	392			
	Between-subject	Group	.175	1	.031	.860	.001
		Error	311.735	56			

*Note: * p ≤ .05*

Table 4: *Immediate (i.e., pre- versus post-measurement) repeated measures ANOVA results for the Update condition of the N-back task, Switch condition of the Switching task, Incongruent condition of the Stroop task, and Stop condition of the Stop-signal task.*

Task condition	Outcome	Factor	Source	Sum of Squares	*df*	*F*	*p*	η_p^2
Update	AC	Within-subject	Time	.145	1	22.148	< .001*	.283
			Time * Group	.001	1	.123	.727	.002
			Error	.367	56			
		Between-subject	Group	.024	1	.598	.443	.011
			Error	2.283	56			
	RT	Within-subject	Time	29132.570	1	7.627	.008*	.120
			Time * Group	3176.680	1	.832	.366	.015
			Error	213903.723	56			
		Between-subject	Group	61211.948	1	2.743	.103	.047
			Error	1249631.241	56			
	RTV	Within-subject	Time	510.784	1	.022	.883	< .001
			Time * Group	55026.948	1	2.374	.129	.041
			Error	1297784.667	56			
		Between-subject	Group	38919.367	1	1.798	.185	.031
			Error	1211928.063	56			
Switch	AC	Within-subject	Time	.495	1	41.460	< .001*	.425
			Time * Group	.006	1	.494	.485	.009
			Error	.668	56			
		Between-subject	Group	.001	1	.009	.923	< .001
			Error	3.236	56			
	RT	Within-subject	Time	903683.153	1	36.683	< .001*	.396
			Time * Group	2732.983	1	.111	.740	.002
			Error	1379552.023	56			
		Between-subject	Group	129009.793	1	1.153	.288	.020
			Error	6265904.193	56			
	RTV	Within-subject	Time	1976.675	1	.047	.830	.001
			Time * Group	3226.251	1	.076	.784	.001
			Error	2376344.967	56			
		Between-subject	Group	206453.271	1	4.524	.038*	.075
			Error	2555314.121	56			
Incongruent	AC	Within-subject	Time	.023	1	7.113	.010*	.113
			Time * Group	< .001	1	.040	.843	.001
			Error	.179	56			
		Between-subject	Group	.013	1	.773	.383	.014
			Error	.944	56			
	RT	Within-subject	Time	72250.708	1	28.787	< .001*	.340
			Time * Group	10.256	1	.004	.949	< .001
			Error	140550.016	56			
		Between-subject	Group	9233.359	1	.482	.490	.009
			Error	1072139.261	56			
	RTV	Within-subject	Time	55671.251	1	4.976	.030*	.082
			Time * Group	792.181	1	.071	.791	.001
			Error	626476.487	56			
		Between-subject	Group	294.610	1	.036	.851	.001
			Error	461939.277	56			
Stop	SSRT	Within-subject	Time	7010.289	1	3.996	.050*	.067
			Time * Group	4927.293	1	2.809	.099	.048
			Error	98243.563	56			
		Between-subject	Group	885.184	1	.173	.679	.003
			Error	286043.982	56			

*Note: AC = accuracy in %, RT = reaction time in ms, RTV = RT variability in ms, SSRT = stop-signal reaction time. * p ≤ .05*

Table 5: *Long-term (i.e., pre- versus six month follow-up measurement) repeated measures ANOVA results for the Update condition of the N-back task, Switch condition of the Switching task, Incongruent condition of the Stroop task, and Stop condition of the Stop-signal task.*

**Task condition**	**Outcome**	**Factor**	**Source**	**Sum of Squares**	***df***	***F***	***p***	η_p^2
Update	AC	Within-subject	Time	.047	1	6.808	.014*	.185
			Time * Group	.006	1	.800	.378	.026
			Error	.207	30			
		Between-subject	Group	< .001	1	.008	.928	< .001
			Error	1.280	30			
	RT	Within-subject	Time	23623.739	1	3.862	.059	.114
			Time * Group	26973.407	1	4.410	.044*	.128
			Error	183512.319	30			
		Between-subject	Group	130210.204	1	6.991	.013*	.189
			Error	558771.195	30			
	RTV	Within-subject	Time	1276.826	1	.067	.798	.002
			Time * Group	21.236	1	.001	.974	< .001
			Error	574891.442	30			
		Between-subject	Group	633.707	1	.046	.832	.002
			Error	415314.862	30			
Switch	AC	Within-subject	Time	.292	1	25.136	< .001*	.456
			Time * Group	.006	1	.545	.466	.018
			Error	.348	30			
		Between-subject	Group	.029	1	.531	.472	.017
			Error	1.612	30			
	RT	Within-subject	Time	179806.538	1	2.918	.098	.089
			Time * Group	914.262	1	.015	.904	< .001
			Error	1848762.684	30			
		Between-subject	Group	136393.269	1	1.511	.229	.048
			Error	2708579.135	30			
	RTV	Within-subject	Time	135708.045	1	2.767	.107	.084
			Time * Group	4388.236	1	.089	.767	.003
			Error	1471374.983	30			
		Between-subject	Group	29203.536	1	.471	.498	.015
			Error	1861037.343	30			
Incongruent	AC	Within-subject	Time	.062	1	11.835	.002*	.283
			Time * Group	< .001	1	.015	.904	< .001
			Error	.158	30			
		Between-subject	Group	.023	1	1.953	.173	.061
			Error	.347	30			
	RT	Within-subject	Time	70265.734	1	15.931	< .001*	.347
			Time * Group	731.232	1	.166	.687	.005
			Error	132314.743	30			
		Between-subject	Group	7706.535	1	.454	.505	.015
			Error	508996.069	30			
	RTV	Within-subject	Time	39753.655	1	7.308	.011*	.196
			Time * Group	24188.305	1	4.446	.043*	.129
			Error	163196.555	30			
		Between-subject	Group	18230.531	1	1.158	.290	.037
			Error	472162.121	30			
Stop	SSRT	Within-subject	Time	3308.476	1	.987	.328	.032
			Time * Group	1804.764	1	.538	.469	.018
			Error	100549.742	30			
		Between-subject	Group	344.807	1	.066	.799	.002
			Error	156185.026	30			

*Note: AC = accuracy in %, RT = reaction time in ms, RTV = RT variability in ms, SSRT = stop-signal reaction time. * p ≤ .05*

Table 6: *Immediate (i.e., pre- versus post-measurement) repeated measures ANOVA results for the power 100 to 500 ms after stimulus onset in the Update condition of the N-back task, Switch condition of the Switching task, Incongruent condition of the Stroop task, and Stop condition of the Stop-signal task.*

**Task condition**	**Factor**	**Source**	**Sum of Squares**	***df***	***F***	***p***	η_p^2
Update	Within-subject	Time	.208	1	.313	.578	.006
		Time * Group	1.601	1	2.407	.127	.046
		Error	33.255	50			
	Between-subject	Group	2.304	1	.875	.354	.017
		Error	131.693	50			
Switch	Within-subject	Time	.460	1	.349	.557	.008
		Time * Group	.042	1	.032	.858	.001
		Error	59.213	45			
	Between-subject	Group	2.155	1	1.355	.250	.029
		Error	71.550	45			
Incongruent	Within-subject	Time	2.867	1	3.701	.060	.064
		Time * Group	.022	1	.029	.866	.001
		Error	41.838	54			
	Between-subject	Group	3.224	1	1.132	.292	.021
		Error	153.744	54			
Stop	Within-subject	Time	.927	1	.861	.359	.020
		Time * Group	3.394	1	3.155	.083	.068
		Error	46.249	43			
	Between-subject	Group	1.766	1	.326	.571	.008
		Error	233.096	43			

*Note: * p ≤ .05*

Table 7: *Long-term (i.e., pre- versus six months follow-up measurement) repeated measures ANOVA results for the power 100 to 500 ms after stimulus onset in the Update condition of the N-back task, Switch condition of the Switching task, Incongruent condition of the Stroop task, and Stop condition of the Stop-signal task.*

**Task condition**	**Factor**	**Source**	**Sum of Squares**	***df***	***F***	***p***	η_p^2
Update	Within-subject	Time	.082	1	.124	.728	.005
		Time * Group	.752	1	1.134	.297	.042
		Error	17.252	26			
	Between-subject	Group	.755	1	.311	.582	.012
		Error	63.103	26			
Switch	Within-subject	Time	.023	1	.012	.915	.001
		Time * Group	.398	1	.200	.659	.009
		Error	43.819	22			
	Between-subject	Group	.583	1	.204	.656	.009
		Error	62.700	22			
Incongruent	Within-subject	Time	7.829	1	7.003	.013*	.200
		Time * Group	.619	1	.554	.463	.019
		Error	31.302	28			
	Between-subject	Group	3.419	1	.933	.342	.032
		Error	102.587	28			
Stop	Within-subject	Time	.140	1	.129	.723	.006
		Time * Group	.001	1	.001	.979	< .001
		Error	21.655	20			
	Between-subject	Group	3.168	1	.501	.487	.024
		Error	126.553	20			

Table 8: Immediate *(i.e., pre- versus post-measurement) repeated measures ANOVA results for the outcomes of the BRIEF-A questionnaire.*

**Outcome**	**Factor**	**Source**	**Sum of Squares**	***df***	***F***	***p***	η_p^2
Total score	Within-subject	Time	1123.457	1	19.497	< .001*	.258
		Time * Group	126.216	1	2.190	.144	.038
		Error	3226.828	56			
	Between-subject	Group	11.802	1	.018	.893	< .001
		Error	35916.207	56			
Working memory	Within-subject	Time	16.690	1	7.863	.007*	.123
		Time * Group	12.448	1	5.865	.019*	.095
		Error	118.862	56			
	Between-subject	Group	4.172	1	.298	.587	.005
		Error	784.379	56			
Shift	Within-subject	Time	24.216	1	10.915	.002*	.163
		Time * Group	1.043	1	.470	.496	.008
		Error	124.241	56			
	Between-subject	Group	2.491	1	.207	.651	.004
		Error	672.724	56			
Task monitor	Within-subject	Time	9.966	1	7.287	.009*	.115
		Time * Group	3.448	1	2.521	.118	.043
		Error	76.586	56			
	Between-subject	Group	< .001	1	< .001	1.000	< .001
		Error	506.862	56			
Inhibit	Within-subject	Time	5.828	1	2.307	.134	.040
		Time * Group	1.690	1	.669	.417	.012
		Error	141.483	56			
	Between-subject	Group	3.448	1	.175	.677	.003
		Error	1100.310	56			

*Note: * p ≤ .05*

Table 9: *Long-term (i.e., pre- versus six months follow-up measurement) repeated measures ANOVA results for the outcomes of the BRIEF-A questionnaire.*

**Outcome**	**Factor**	**Source**	**Sum of Squares**	***df***	***F***	***p***	η_p^2
Total score	Within-subject	Time	4691.338	1	48.168	< .001*	.566
		Time * Group	15.236	1	.156	.695	.004
		Error	3603.611	37			
	Between-subject	Group	18.776	1	.025	.876	< .001
		Error	28290.942	37			
Working memory	Within-subject	Time	72.806	1	23.317	< .001*	.387
		Time * Group	2.857	1	.915	.345	.024
		Error	115.528	37			
	Between-subject	Group	12.025	1	.856	.361	.023
		Error	519.591	37			
Shift	Within-subject	Time	32.336	1	12.483	.001*	.252
		Time * Group	1.105	1	.427	.518	.011
		Error	95.843	37			
	Between-subject	Group	11.625	1	1.171	.286	.031
		Error	367.170	37			
Task monitor	Within-subject	Time	57.721	1	43.887	< .001*	.543
		Time * Group	.286	1	.217	.644	.006
		Error	48.663	37			
	Between-subject	Group	2.143	1	.194	.662	.005
		Error	408.242	37			
Inhibit	Within-subject	Time	23.636	1	10.081	.003*	.214
		Time * Group	.046	1	.020	.889	.001
		Error	86.749	37			
	Between-subject	Group	1.081	1	.052	.820	.001
		Error	762.791	37			

*Note: * $p \leq .05$*
